# Bilateral Corrective Mandibular Ostectomy—A Salvage Technique for Traumatic Masticatory Fibrosis in a Dog

**DOI:** 10.3390/vetsci12060531

**Published:** 2025-05-30

**Authors:** Leann Shaw, Darryl Millis

**Affiliations:** 1 VCA California Veterinary Specialists, Murrieta, CA 92563, USA; leannshawdvm@gmail.com; 2 Department of Small Animal Clinical Sciences, College of Veterinary Medicine, University of Tennessee, Knoxville, TN 37996, USA

**Keywords:** muscle fibrosis, masticatory muscles, mandibular ostectomy, muscle fibrosis

## Abstract

End-stage masticatory muscle fibrosis may occur following trauma, resulting in the inability to open the mouth for eating, drinking, panting and vomiting. Following MRI and EMG confirmation of end-stage muscle fibrosis, a bilateral wedge ostectomy was performed of the mandibles, leaving a permanent mouth opening of 3.5 cm. The osteotomies were stabilized with 6-hole dynamic compression plates and screws. A temporary percutaneous endoscopic gastrotomy tube was placed while the dog underwent rehabilitation and reconditioning. The dog was able to eat, drink, and pant after recovery, which allowed the dog to live a relatively normal and good quality life. Wedge osteotomies of the mandibles with bone plate stabilization may allow dogs with permanent restrictive mouth opening due to fibrosis of the masticatory muscles to live a relatively normal life.

## Simple Summary

Restrictive mouth opening (RMO) in dogs can occur following jaw trauma that results in masticatory muscle trauma and end-stage muscle fibrosis. An 8-year-old intact male German Shepherd presented for RMO after playing with a large ball that was lodged in the mouth. Muscle atrophy and a progressive inability to open the mouth ensued over the following weeks, requiring syringe feedings for nutrition. Magnetic Resonance Imaging (MRI) demonstrated severe muscle atrophy, and electromyography (EMG) activity suggested a loss of masticatory muscle function. A surgical salvage procedure consisting of a bilateral mandibular wedge ostectomy was performed with bone plates to stabilize the mandibles. This allowed the mouth to remain permanently open, allowing the dog to perform life-sustaining functions, including the ability to eat, drink, pant, and vomit. The patient recovered and was able to function relatively normally with a good quality of life. This salvage procedure is a treatment option for permanent fibrosis of the masticatory muscles in dogs.

## 1. Introduction

Difficulty opening the jaw, or trismus, is relatively uncommon in dogs. Immune-mediated masticatory muscle myositis is one of the more common causes that results in pain or difficulty opening the mouth, in which autoantibodies to type 2M muscle fibers are formed [1]. Treatment generally involves immunosuppressive drug therapy early in the course of the disease, and continuing for several months [2,3,4]. Other causes of restrictive mouth opening (RMO), or an inability to open the jaw, include fusion of the temporomandibular joint or healed fractures [1]. Patients that do not receive timely treatment of masticatory muscle myositis may have fibrosis of the masticatory muscles and trismus [5,6]. This condition has been treated with the forcible opening of the jaw under anesthesia, followed by physical rehabilitation to try to maintain varying degrees of a normal range of motion. This approach carries significant morbidity, including temporomandibular joint luxation and mandibular fractures with no documented long-term clinical improvement, and is not recommended [1,3]. Therefore, severe fibrosis of the masticatory muscles due to immune-mediated or other causes may benefit from a different approach.

In this case report, we describe osteotomies of the mandible to treat presumed trauma-related fibrosis of the muscles of mastication and trismus as a successful approach to returning the dog to relatively normal function. We are unaware of any studies using surgical management for severely affected cases.

## 2. Case Presentation

### 2.1. History and Physical Exam Findings

An 8-year-old 28 kg intact male German Shepherd was evaluated because of RMO. He was originally seen at a veterinary hospital after playing with a large ball that became lodged in the mouth for approximately 30 min. Initially, a dropped jaw occurred, and exophthalmos developed, presumably due to masticatory muscle trauma and possible trigeminal nerve neuropraxia. He was treated with methocarbamol, carprofen, and supportive care for 30 days. Temporal and masseter muscle atrophy occurred with a progressive inability to open the jaw over a period of 2 weeks. The dog was unable to open its mouth because of masticatory muscle fibrosis, and syringe feedings were necessary to provide nutrition and water. Medications were adjusted to include prednisone and gabapentin. Because recovery did not occur and a 9 kg weight loss was observed, a gastrotomy tube was placed to allow alimentation. Despite the placement of the gastrotomy, the patient did not gain weight, and the owner was concerned about the volume and frequency of tube feedings and the dog’s quality of life. An additional concern of trying to increase caloric intake was placing too much liquid into the stomach, with the risk of nausea, vomiting, and potential aspiration pneumonia. The patient was referred to discuss other alternatives aside from tube feedings or total parenteral nutrition, both of which would not be viable long-term solutions based on the owner’s goals.

**Figure 1:**
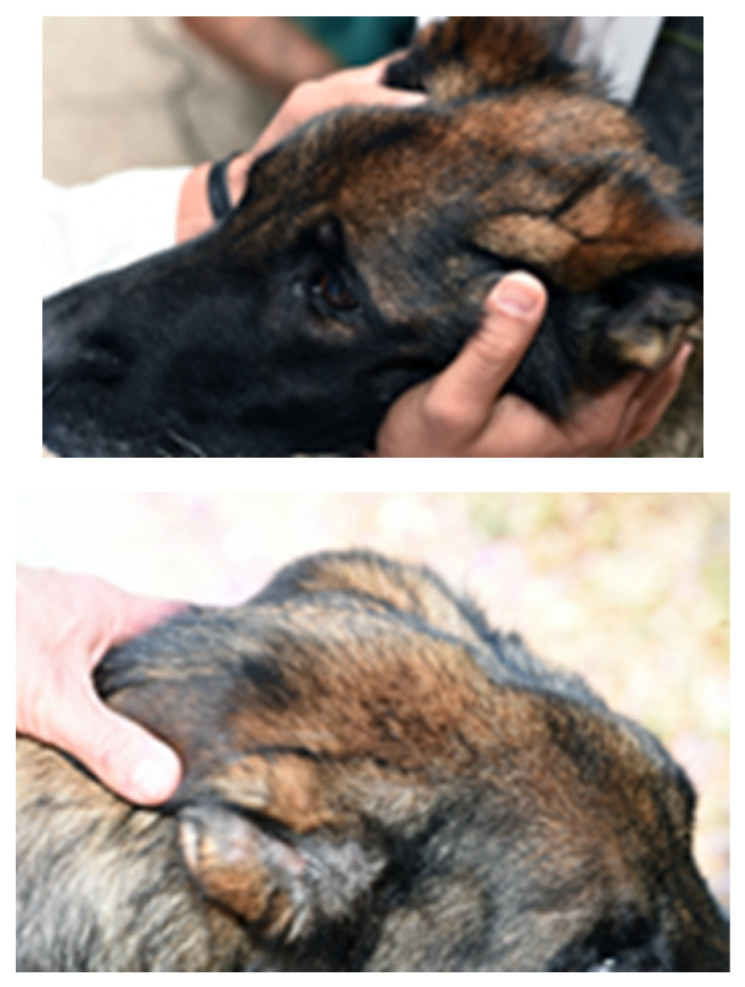

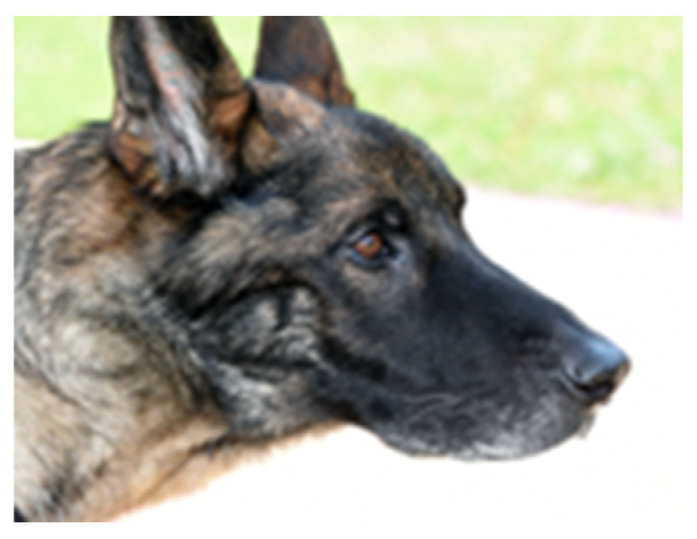
Photographs of an 8-year-old 28 kg (63 lbs) intact male German Shepherd presenting for trauma to masticatory muscles. Masticatory muscle fibrosis and restricted mouth opening resulted in weight loss.

**Figure 2:**
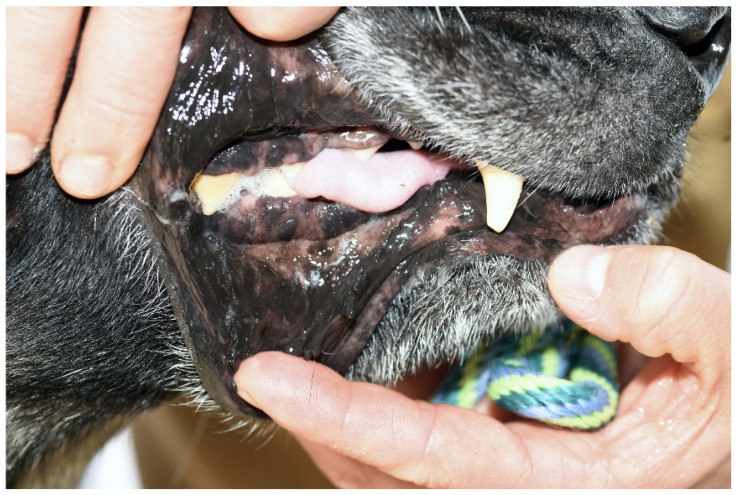
Physical examination of the oral cavity demonstrated restrictive mouth opening with complete inability to open the jaw.

On presentation to the University of Tennessee College of Veterinary Medicine, the patient was thin (BCS 3/9); other vital signs were within normal limits. The original trauma resulted in permanent damage to muscles of mastication, with resultant atrophy and fibrosis of the masticatory muscles. (Figure 1 and Figure 2). No pain was elicited on manipulation or palpation and the mouth could not be opened.

### 2.2. Diagnostic Imaging Findings and Interpretation

**Figure 3:**
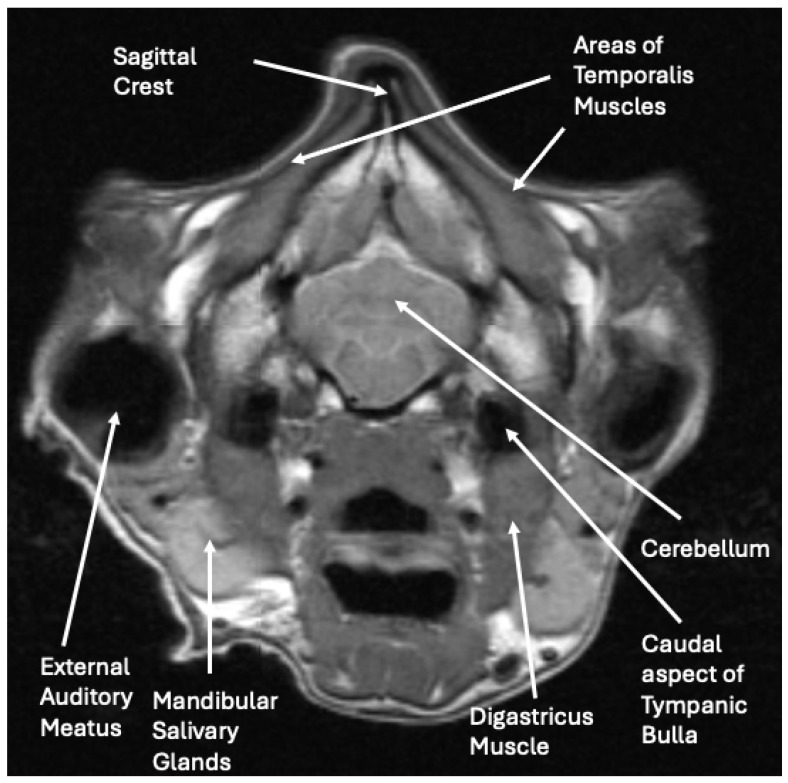
Cross-sectional MRI image at the level of the caudal cerebellum, internal auditory canal, and middle ear, demonstrating severe atrophy of the temporalis muscles.

The radiographs and abdominal ultrasound performed at the previous referral hospital revealed no significant systemic disease findings. The MRI images indicated severe atrophy of the temporal, masseter, and pterygoid muscles bilaterally with multifocal ring enhancement and focal mineralization or hemorrhage of the right temporal muscle (Figure 3). Muscle denervation, necrosis, and fibrosis secondary to the reported traumatic event with suspected vascular and innervation compromise were considered the most likely cause for the changes noted on the MRI. The EMG activity of the masticatory muscles bilaterally was silent, suggesting diffuse fibrosis and a loss of function. The pre-operative radiographs demonstrated normal skeletal structures.

### 2.3. Treatment

The owner was offered and elected a salvage surgical correction in attempts to provide a better quality of life. Prior to surgery, planning regarding the location of the ostectomies and the size of the wedge to be removed were calculated from radiographs and the use of a similar-sized mandible. After fasting prior to surgery, the dog was premedicated with methadone (0.5 mg/kg), mg acepromazine (0.01 mg/kg), and maropitant (1 mg/kg) IV. Anesthesia was induced with ketamine (5 mg/kg) and mg midazolam (0.5 mg/kg) IV, and the dog was maintained with isoflurane inhalant in 100% oxygen for the surgical procedure. Cefazolin (22 mg/kg) was administered IV every 2 h perioperatively. A dose of 0.8 mL of Septicaine was injected locally in the region of both mandibular canals for additional pain control, along with a fentanyl (5 mcg/kg, 50 mcg/mL) and lidocaine (2 mg/kg, 100mg/mL) bolus IV. A fentanyl/lidocaine continuous rate infusion was continued at 12.4 mL/h for pain control.

A temporary tracheostomy was created, and a tracheostomy tube was placed to maintain adequate anesthesia during the surgical procedure. The tracheostomy tube was removed post-operatively and the stoma was allowed to heal by second intention.

**Figure 4:**
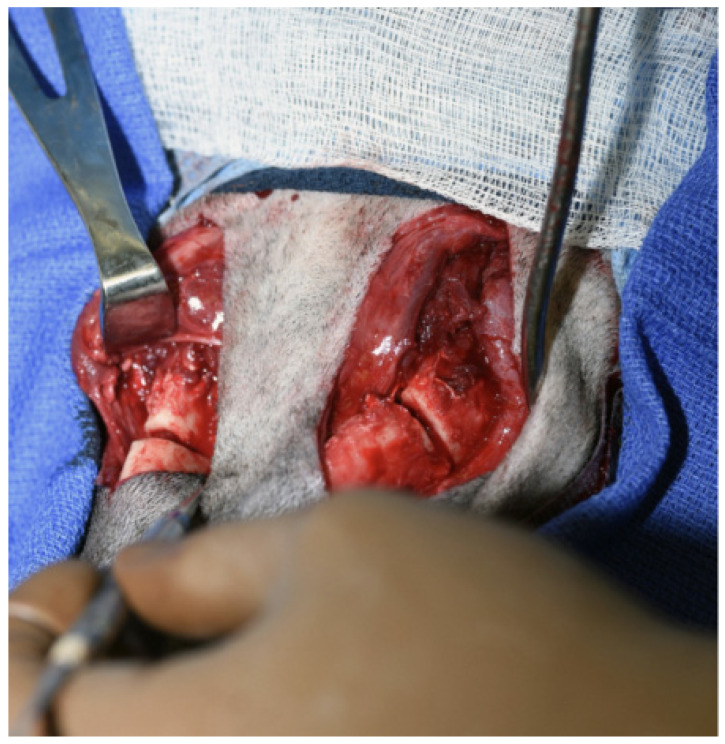
A surgical incision was made on either side of the mandibles and a bilateral mandibular wedge ostectomy was performed using an oscillating bone saw 2 cm rostral to the border of the ventral ramus.

**Figure 5:**
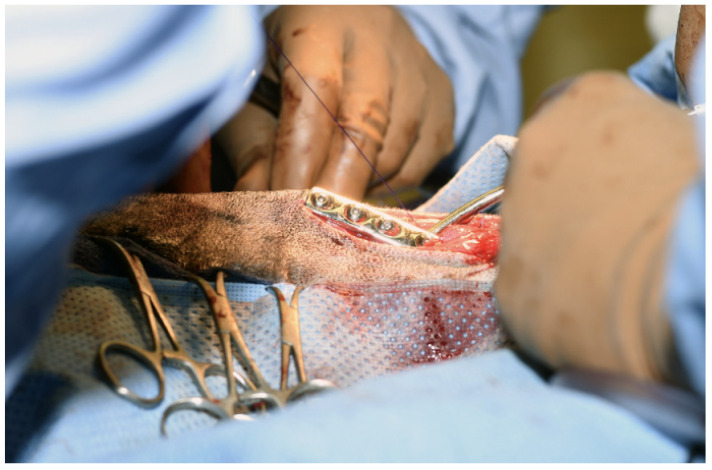
A 6-hole, 3.5 mm compression plate was placed on the lateral aspect of each mandibular body.

**Figure 6:**
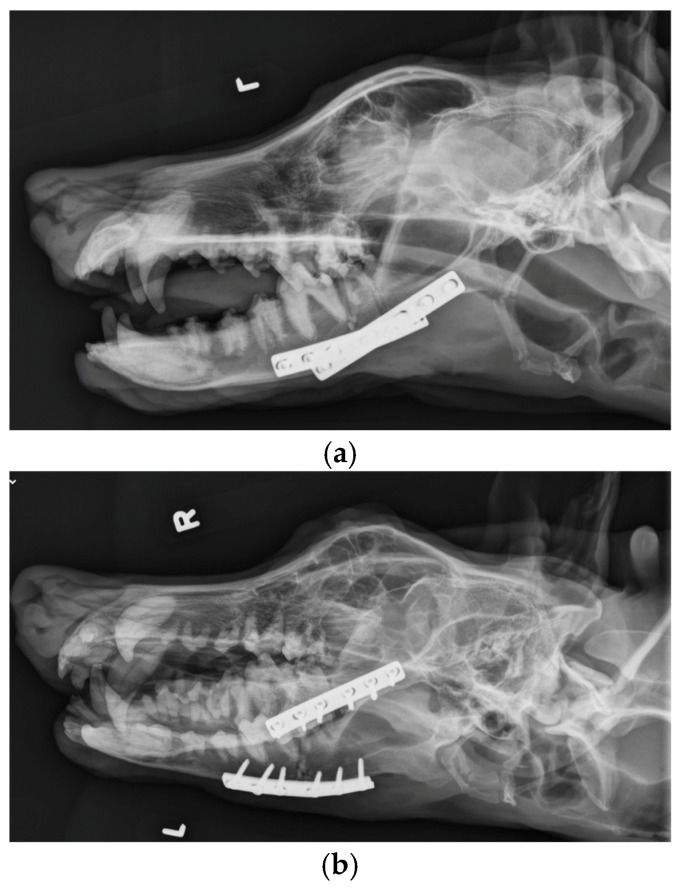

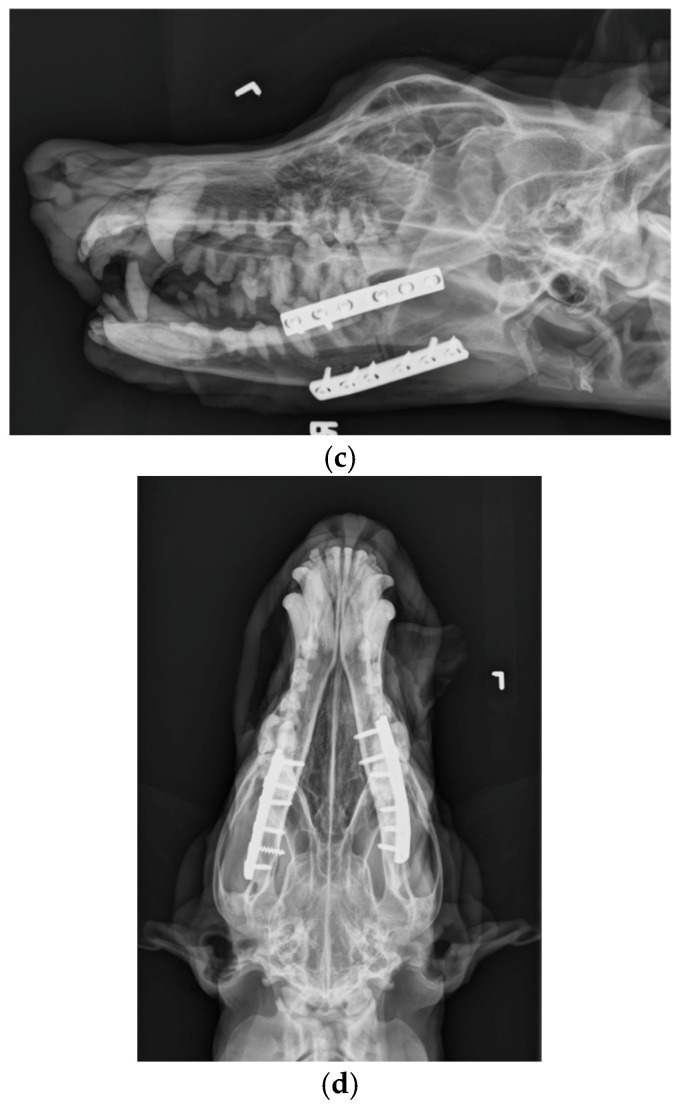
(**a**). Lateral. (**b**). Right dorsal-left ventral oblique. (**c**). Left dorsal-right ventral oblique. (**d**). Ventrodorsal view demonstrating bone plates and screws applied to the mandibular ramus bilaterally.

**Figure 7:**
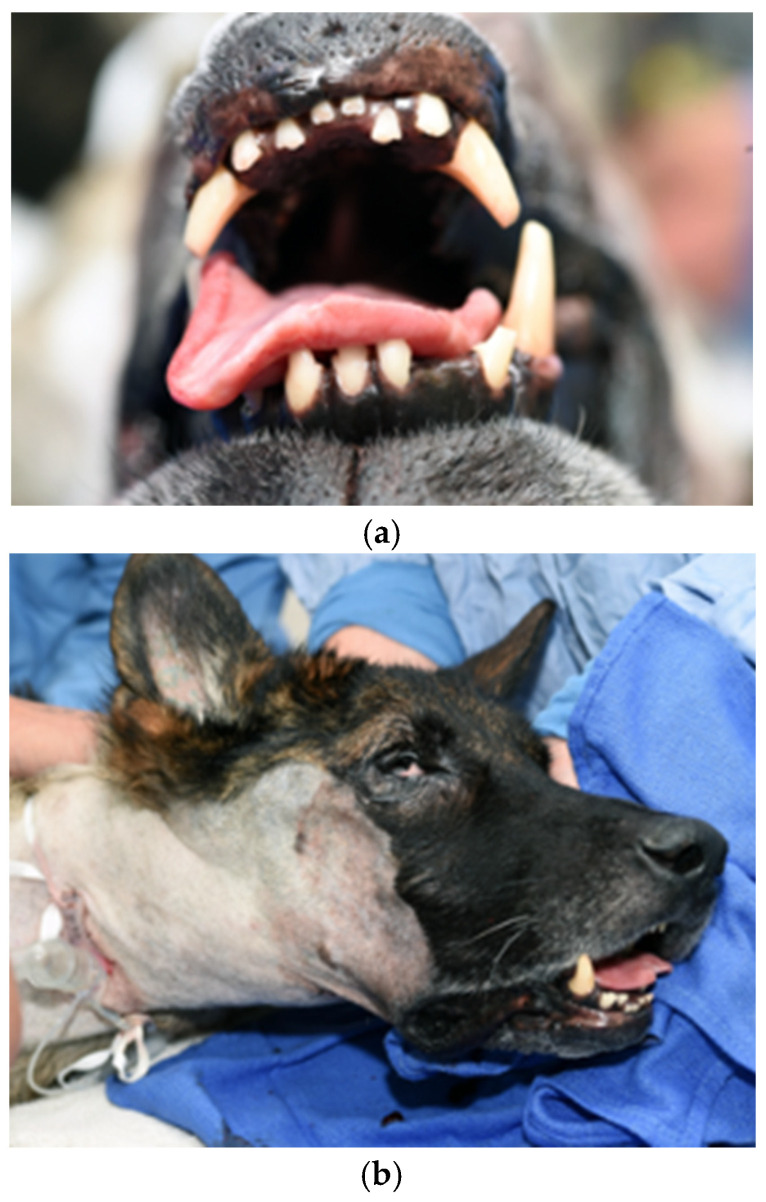
(**a**). Immediate postoperative photo of oral opening. The patient is in dorsal recumbency. (**b**). Appearance of patient immediately postoperatively while recovering from anesthesia.

The patient was placed in dorsal recumbency and aseptically prepped from the level of the second tracheal ring to the rostral mandible and laterally to the ventral border of the zygomatic arch. An incision was made bilaterally over the caudal two-thirds of the ventral border of the mandibles and through the platysma muscle for exposure of the periosteum. The myloglossus and digastricus muscles were elevated from their attachments on the bone. A bilateral mandibular wedge ostectomy was performed, and bone plates and screws were used on either side to stabilize the mandibles to allow the mouth to remain permanently open to allow drinking, eating, and panting. (Figure 4 and Figure 5). The proposed ostectomy was outlined on the lateral and medial surfaces of each mandible. An oscillating bone saw was used to perform osteotomies along the medial and lateral aspects of each mandible, avoiding the mandibular canal. An osteotome was used to separate any remaining attachments between bones. The oral opening was visualized by a nonsterile assistant, and a spacer was placed to maintain an opening of approximately 3.5 cm. Bone was removed with the sagittal saw to create a closing wedge ostectomy to maintain the desired oral opening and maintain cortical contact of the mandible. Six-hole, 3.5 mm dynamic compression plates were placed on the ventrolateral aspect of the right and left mandibles with self-tapping, cortical screws to oppose the ostectomy sites, taking care to avoid tooth roots. Cancellous bone from the removed bone wedges was placed at the ostectomy lines. A minor operative complication was left mandibular artery trauma with hemorrhage that was controlled with bone wax. The post-operative radiographs confirmed both reduction and stabilization of the mandibular ramus bilaterally by means of the two plates. The tooth roots were avoided with screw placement (Figure 5 and Figure 6). The 3.5 cm opening was maintained between the upper and lower incisors postoperatively (Figure 7).

A low-profile percutaneous endoscopic gastrotomy (PEG) tube was inserted to replace the previously placed gastrotomy tube. The post-operative radiographs demonstrated proper placement. The tube was inserted to provide temporary supplemental feedings while the dog learned to use and strengthen his tongue.

Recovery was uneventful. The dog was maintained on a fentanyl (5 mcg/kg, 50 mcg/mL/h) and lidocaine (2 mg/kg/h, 100 mg/mL) CRI for the first 72 h and Plasmalyte IV fluids at a maintenance rate. The mandible was iced for 15 min every 6 h for 48 h post-operatively. Post-operative medications included amoxicillin–clavulanic acid (375 mg q 12 h), gabapentin (1200 mg q 8 h), and carprofen (100 mg q 12 h).

### 2.4. Outcome and Clinical Relevance

Following surgery, the dog was able to use his tongue to lap small amounts of liquefied food and water. Additional feedings were supplied through the PEG tube via a slurry diet mixture initially at resting energy requirements. Over the next several days, both the caloric and protein intake were increased to aid with muscle mass gain. At-home rehabilitation exercises included small amounts of peanut butter smeared to the roof of the mouth or in a toy to exercise the tongue. The patient recovered, gained weight to achieve normal body condition score (BCS 5/9), and was able to function relatively normally with a good quality of life until it was euthanized for unrelated causes 5 years after surgery.

## 3. Discussion

To the authors’ knowledge, there are no published reports regarding salvage procedures to treat permanent fibrosis of the masticatory muscles in dogs. Most cases of masticatory muscle conditions in dogs are associated with immune-mediated masticatory myositis [2,3,4]. These are generally recognized and treated with immunosuppressive therapy and do not reach end-stage fibrosis. In human medicine, the condition of inflamed or contracted muscles of mastication (masseter, lateral pterygoid, medial pterygoid, and temporal muscles) causing restricted mouth opening is classified as trismus. Traumatic inciting causes include oral surgery, temporomandibular joint disorders, hyperplasia of the coronoid process, radiation therapy, infection, and other traumas [7,8]. Typical treatment involves the forced opening of the mouth followed by physical therapy. Alternatively, the syndrome myositis ossificans describes heterotopic bone formation within a muscle [9]. This is further classified into two separate groups: myositis ossificans progressiva, a genetic autosomal dominant disease, and myositis ossificans traumatica (MOT), occurring because of repeated trauma [10]. The MRI findings in our case suggest mineralization in the muscles of mastication, suggesting that this may be MOT. MOT is considered a rare syndrome of the masticatory muscles causing trismus in humans. Treatment of MOT includes surgical excision of the affected tissue followed by physical therapy. Finally, masticatory muscle tendon–aponeurosis hyperplasia may also result in RMO in humans. A bilateral coronoidectomy and/or coronoidotomy and anglectomy may be performed for this condition. Based on the severe nature of our case, we do not believe that these procedures would have been adequate to improve function of the patient.

Dynamic compression plates were used in this case to provide rigid internal fixation, without entering the oral cavity. Because osteotomies were created, fragment surfaces could be compressed to provide additional stability. Recommendations have been made to use two plates, one to act as a tension plate and the other on the ventral surface to provide additional stability [11]. However, in this case, the jaw was unable to be opened, and there was no remaining muscle to initiate chewing. Therefore, we believed that plating each side would provide adequate stability for bone healing. Locking plates and screws would potentially add more stability [11], but because of the cost and the fact that failure would be less likely in a dog unable to move the jaw, dynamic compression plates were used. The complications of mandible fractures have been reviewed [11,12], and include infection, damage to the tooth roots, and malocclusion. Because we performed an ostectomy, we were able to avoid communication with the oral cavity, which should reduce the chances of implant-associated infection. Osteomyelitis was much more common in dogs with open factures compared with closed fractures in one study [13]. In fact, there was no indication of infection during the 5-year follow-up in this patient, although communication was limited to infrequent phone communication with the owner and attending veterinarian. One caution is to be aware of the possibility of dental tartar and periodontal disease is these cases due to altered function. This case did require regular dental care. We took care to avoid damage to tooth roots during the osteotomies and implant placement. Although tooth root damage is well recognized in dogs with mandibular fractures, there was no indication that it occurred in this patient. Ventral plate placement and a careful ostectomy using a sagittal saw with an osteotome to complete the ostectomy may help to reduce tooth root damage and damage to the mandibular artery. Although malocclusion has been reported as the most common complication in studies [12,13], this was not an issue in this patient because the mouth was fixed in an open position.

In dogs, RMO results in inability to open the mouth to perform life-sustaining activities, such as eating, drinking, vomiting, or panting. Although uncommon, in dogs with permanent muscle fibrosis or nerve injury, the bilateral corrective mandibular ostectomy surgery described here is an option as a salvage procedure. The results of this salvage procedure in this patient were acceptable and facilitated a return to normal intake of nutrition and quality of life.

## 4. Conclusions

End-stage fibrosis of the muscles of mastication can be devastating, resulting in an inability to eat or drink without other means of alimentation. Additionally, severe trismus may result in life-threatening consequences if the dog is unable to vomit or pant. The salvage procedure described here of creating osteotomies of the mandibles to permanently create a fixed oral opening may allow dogs to perform life-sustaining functions and lead a relatively good quality of life and should be considered in dogs with restrictive mouth opening. As further cases are encountered and published, refinement will occur in both surgical techniques and quality of life outcomes.

## Data Availability

No new data were created or analyzed in this study.

## References

[1] Melmed C., Shelton G.D., Bergman R., Barton C. (2004). Masticatory Muscle Myositis: Pathogenesis, Diagnosis, and Treatment. Compend. Cont. Educ. Pract. Vet..

[2] Castejon-Gonzalez A.C., Soltero-Rivera M., Brown D.C., Reiter A.M. (2018). Treatment Outcome of 22 Dogs with Masticatory Muscle Myositis (1999–2015). J. Vet. Dent..

[3] Gilmour M., Morgan R., Moore F. (1992). Masticatory Myopathy in the Dog: A Retrospective Study in 18 Cases. J. Amer. Anim. Hosp. Assoc..

[4] Foreman M., Cherubini G.B. (2021). Dexamethasone Can Be Safely and Effectively Used for Treatment of Masticatory Muscle Myositis in Dogs. Top. Companion Anim. Med..

[5] Reed F., Iff I. (2012). Case Report Rapport de Cas Use of a Laryngeal Mask Airway in a Brachycephalic Dog with Masticatory Myositis and Trismus. Can. Vet. J..

[6] Nanai B., Phillips L., Christiansen J., Shelton G.D. (2009). Life Threatening Complication Associated with Anesthesia in a Dog with Masticatory Muscle Myositis. Vet. Surg..

[7] Bijal A., Chandra S.B. (2019). Recognizing Trismus Symptoms, Prevention and Treatment. Med-Science.

[8] Yoshida H., Oshiro N., Fukuda A., Gamoh S., Shimizutani K., Morita S. (2014). A Case of Reformed Coronoid Process and Mandibular Angle after Coronoidectomy and Anglectomy for Masticatory Muscle Tendon-Aponeurosis Hyperplasia. Oral Radiol..

[9] Aoki T., Naito H., Ota Y., Shiiki K. (2002). Myositis Ossificans Traumatica of the Masticatory Muscles: Review of the Literature and Report of a Case. J. Oral Maxillofac. Surg..

[10] Hanisch M., Hanisch L., Fröhlich L.F., Werkmeister R., Bohner L., Kleinheinz J. (2018). Myositis Ossificans Traumatica of the Masticatory Muscles: Etiology, Diagnosis and Treatment. Head Face Med..

[11] Boudrieau R.J., Tobias K.T., Johnston S.A. (2012). Mandibular and Maxillofacial Fractures. Veterinary Surgery: Small Animal.

[12] Wolfs E., Arzi B., Cota J.G., Kass P.H., Verstraete F.J.M. (2022). Craniomaxillofacial trauma in immature dogs—Etiology, treatments, and outcomes. Front. Vet. Sci..

[13] Umphlet R.C., Johnson A.L. (1990). Mandibular fractures in the dog: A retrospective study of 157 cases. Vet. Surg..

